# Endogenous lycopene improves ethanol production under acetic acid stress in *Saccharomyces cerevisiae*

**DOI:** 10.1186/s13068-018-1107-y

**Published:** 2018-04-10

**Authors:** Shuo Pan, Bin Jia, Hong Liu, Zhen Wang, Meng-Zhe Chai, Ming-Zhu Ding, Xiao Zhou, Xia Li, Chun Li, Bing-Zhi Li, Ying-Jin Yuan

**Affiliations:** 1 Key Laboratory of Systems Bioengineering (Ministry of Education), School of Chemical Engineering and Technology, Tianjin University, Tianjin, 300072 People’s Republic of China; 2 SynBio Research Platform, Collaborative Innovation Center of Chemical Science and Engineering (Tianjin), Tianjin University, Tianjin, 300072 People’s Republic of China

**Keywords:** Acetic acid, *Saccharomyces cerevisiae*, Lycopene, Reactive oxidative species

## Abstract

**Background:**

Acetic acid, generated from the pretreatment of lignocellulosic biomass, is a significant obstacle for lignocellulosic ethanol production. Reactive oxidative species (ROS)-mediated cell damage is one of important issues caused by acetic acid. It has been reported that decreasing ROS level can improve the acetic acid tolerance of *Saccharomyces cerevisiae*.

**Results:**

Lycopene is known as an antioxidant. In the study, we investigated effects of endogenous lycopene on cell growth and ethanol production of *S. cerevisiae* in acetic acid media. By accumulating endogenous lycopene during the aerobic fermentation of the seed stage, the intracellular ROS level of strain decreased to 1.4% of that of the control strain during ethanol fermentation. In the ethanol fermentation system containing 100 g/L glucose and 5.5 g/L acetic acid, the lag phase of strain was 24 h shorter than that of control strain. Glucose consumption rate and ethanol titer of yPS002 got to 2.08 g/L/h and 44.25 g/L, respectively, which were 2.6- and 1.3-fold of the control strain. Transcriptional changes of *INO1* gene and *CTT1* gene confirmed that endogenous lycopene can decrease oxidative stress and improve intracellular environment.

**Conclusions:**

Biosynthesis of endogenous lycopene is first associated with enhancing tolerance to acetic acid in *S. cerevisiae*. We demonstrate that endogenous lycopene can decrease intracellular ROS level caused by acetic acid, thus increasing cell growth and ethanol production. This work innovatively   puts forward a new strategy for second generation bioethanol production during lignocellulosic fermentation.

**Electronic supplementary material:**

The online version of this article (10.1186/s13068-018-1107-y) contains supplementary material, which is available to authorized users.

## Background

Lignocellulosic ethanol is routinely recognized as one of the most promising renewable energy sources, and has received widespread attention due to its economic and environmental benefits [1, 2]. However, the inhibitory compounds generated during the pretreatment of lignocellulosic biomass, mainly including furan, weak acids, and phenolic mixtures, have a significant inhibitory effect on cell growth, metabolism, and ethanol production [3–5]. Acid-catalyzed hydrolysis of lignocelluloses usually generates acetic acid as the byproduct [6, 7], which is one of the main toxic inhibitors [8, 9]. Acetic acid results in a decrease in metabolic enzyme activity, cell growth, and ethanol production of *Saccharomyces cerevisiae* [10, 11]. Moreover, it can also induce the accumulation of reactive oxygen species (ROS) [12], resulting in damage to biological macromolecules such as DNA, lipids, and proteins, leading to the loss of protein function and even programmed cell death [13]. Therefore, strengthening the tolerance of fermented microorganisms to acetic acid is an important challenge in the lignocellulosic ethanol production process.

The development of acetic acid-tolerant yeast strains is mainly through reducing the absorption of acetic acid, enhancing the efflux of hydrogen ions and acetate ions, and enhancing the intracellular metabolism of acetic acid. Overexpressing of *AZR1* [14], *ACS2* [15], *HAA1* [16–18], and *HRK1* [18] could reduce the concentration of intracellular acetic acid, respectively. Knockouting of *QDR3* [19] and overexpressing of *WHI2* [20], *SFP1,* and *ACE2* [21] could elicit endogenous acetic acid resistance in *S. cerevisiae,* respectively. Liu et al. [22] found changes in the gene expression in the acetic acid-resistant histone *H3*/*H4* mutants were mainly related to energy production and antioxidative stress. Landolfo et al. [23] found that cellular ROS accumulation and scavenging status can significantly affect cell viability and ethanol production in *S. cerevisiae*. Acetic acid can provoke the oxidative stress of *S. cerevisiae* [12]. Antioxidants, due to their high ability to scavenge intracellular ROS species, have great potential to enhance the proliferation capacity of a broad range of cells. Therefore, adding of antioxidants has become a method of choice for construction of acetic acid-tolerant yeast strain. Qi et al. [24] increased the cell viability and ethanol titer of *P. guilliermondii* by adding biotin in seed cells. Wan et al. [25] proved that zinc addition decreased the release of ROS in the presence of chronic acetic acid stress. Previous study demonstrated that the addition of proline or overexpression of a proline synthesis-related gene (*PRO1*) led to an obvious increase in tolerance to acetic acid [26]; recently, overexpression of a peroxiredoxin in *S. cerevisiae* has showed an enhanced tolerance to lignocellulose-derived inhibitors including acetic acid [27]. Carotenoids are well known as antioxidants for protecting cells and organisms in nature [28, 29]. They  are important biological compounds that can inactivate electronically excited molecules, a process termed quenching. However, it has not been reported that the utilization of carotenoids as antioxidant to protect of *S. cerevisiae* during ethanol fermentation from acetic acid stress.

In this study, the effects of lycopene on cell growth and ethanol production were investigated in *S. cerevisiae* under acetic acid stress. By accumulating lycopene during the aerobic fermentation of the seed stage, the ROS level of yeast was decreased and the ethanol production rate of yeast was increased under acetic acid stress during anaerobic fermentation. This work highlights that endogenous expression of lycopene in yeast can improve cell viability and ethanol production under acetic acid stress. The strategy proposed here may provide a new and alternative direction for the construction of tolerant inhibitor strains.

## Results and discussion

### Expressing endogenous lycopene to decrease intracellular ROS levels

**Fig. 1:**
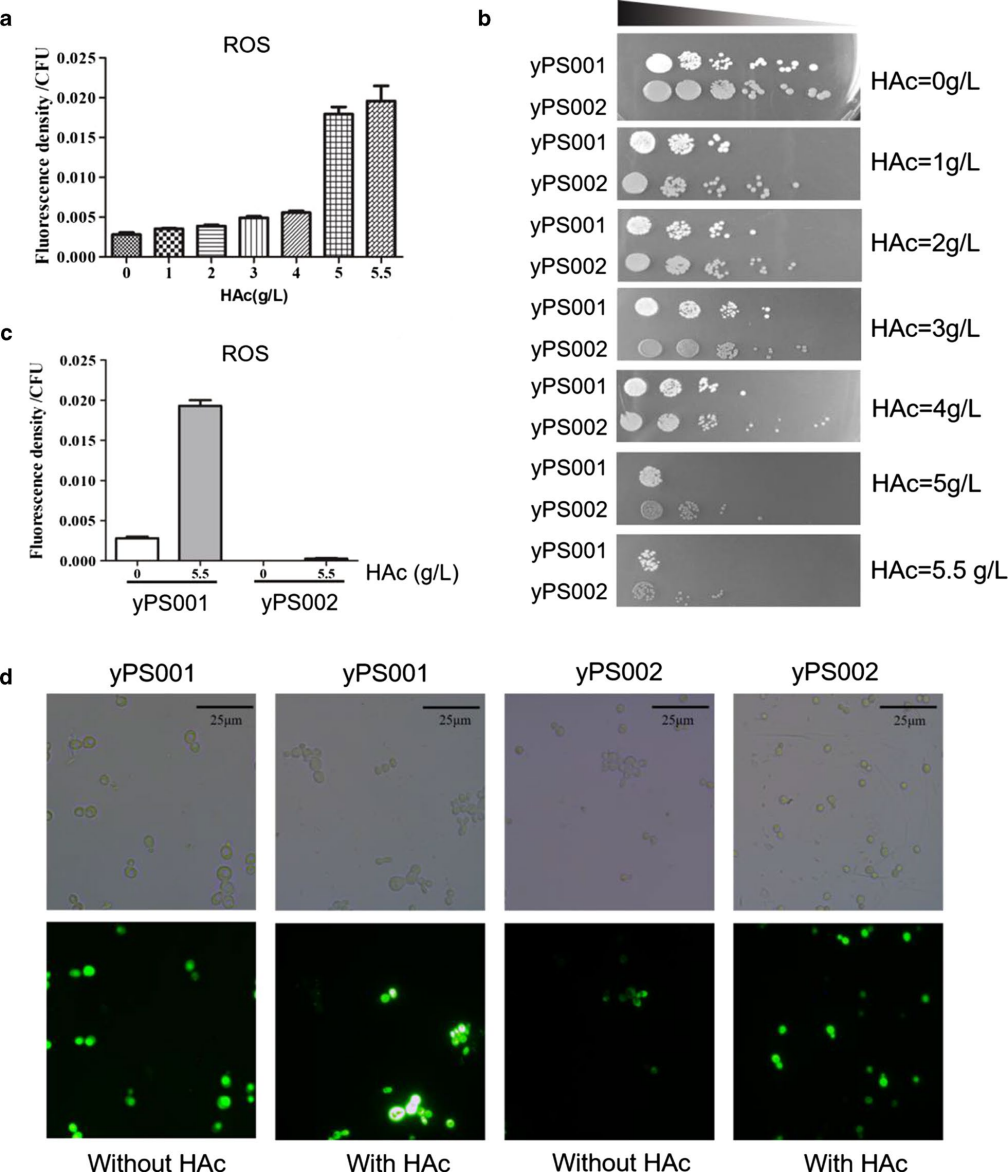
Effects of lycopene on cell growth and ROS accumulation in acetic acid media. **a** Effects of acetic acid on ROS accumulation of CEN.PK2-1C. **b** Growth phenotypes of the yPS001 and yPS002 strains under conditions of acetic acid stress. **c** Effects of acetic acid on ROS levels of yPS001 and yPS002. **d** Fluorescence intensity of yPS001 and yPS002. HAc was abbreviated from acetic acid in figures. Data are averages from three independent experiments (error bars represent SD)

To assay the intracellular ROS levels caused by acetic acid [30], CEN.PK2-1C incubated in YPD media with various concentrations of acetic acid was examined (Fig. 1a). The intracellular ROS levels of the cells incubated in YPD media with 0, 1, 2, 3, 4, 5, and 5.5 g/L of acetic acid were 0.0028, 0.0035, 0.0039, 0.0049, 0.0056, 0.0179, and 0.0196 fluorescence density per cell, respectively. It is demonstrated that the intracellular ROS level was strongly associated with extracellular acetic acid level.

In this study, lycopene production yeast was generated by incorporating the lycopene pathway into CEN.PK2-1C (yPS002). Then, the CEN.PK2-1C with pRS415 was used as control (yPS001). To examine the effect of endogenous expression of lycopene on cell growth, yPS001 and yPS002 were cultured in YPD media until the late log phase, and serially diluted cells were spotted onto YPD agar plates containing 0–5.5 g/L acetic acid. As shown in Fig. 1b, the growth of yPS001 and yPS002 was almost the same in the absence of acetic acid, indicating that the expression of lycopene did not affect the cell growth. However, growth defects were observed in the control strain (yPS001) compared to the lycopene-expressing strain (yPS001) on plates containing 1.0, 2.0, 3.0, and 4 g/L acetic acid. There was a significantly better growth for the yPS002 over the yPS001 on media containing 5.0 and 5.5 g/L acetic acid. These results indicated that the incorporation of lycopene pathway could increase the cell growth in media with acetic acid. Moreover, to demonstrate the versatility of this method in different chassis strains, the lycopene pathway was transferred into BY4741 (yPS009) and BY4742 (yPS011), and the BY4741 and BY4742 with pRS415 were used as control (yPS008, yPS010), respectively. As shown in Additional file 1: Figure S1, significant growth advantages of yPS009 and yPS011 were observed in the presence of acetic acid. The experiment suggested that the lycopene production can be used to increase acetic acid tolerance of yeast in different yeast chassis.

To further explore the mechanism of the observed effects of endogenous lycopene on cell growth under acetic acid, we examined the intracellular ROS levels of yPS001 and yPS002. As shown in Fig. 1c, the intracellular ROS level of yPS002 was notably lower than that of yPS001 in groups of both acetic acid-free and acetic acid-treated. In the group of acetic acid-free, the ROS level of yPS001 was about 289-fold of that of yPS002. Under the condition of 5.5 g/L acetic acid, intracellular ROS levels of the two strains displayed a sharp increase in contrast to that of acetic acid-free group. Treated with 5.5 g/L acetic acid, yPS001 and yPS002 generated sevenfold and 27-fold ROS levels of that in the group of acetic acid-free, which further indicated that acetic acid could indeed cause ROS generating [12]. However, in the group of acetic acid-treated, the intracellular ROS level of yPS002 was just merely 1.4% of that of yPS001. Then, the fluorescence intensity of DCFH treatment was observed (Fig. 1d). The result of fluorescence intensity was consistent with the value of ROS, and the ROS level of yPS002 was indeed lower than that of yPS001 under the same condition.

### Effects of various lycopene production on acetic acid tolerance

**Fig. 2:**
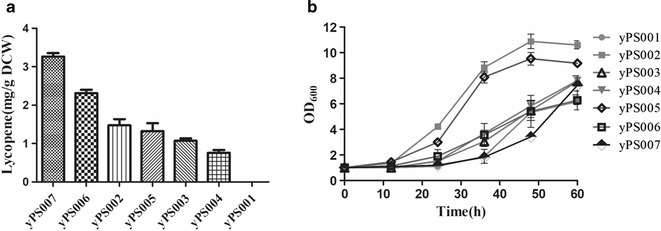
Effects of different lycopene levels on cell growth in yeast. **a** Lycopene production of different strains. **b** Growth curves of yeast containing different lycopene levels during ethanol fermentation (10% glucose, 1% yeast extract, 2% peptone, and 5.5 g/L acetic acid). Data are averages from three independent experiments. (error bars represent SD)

To study the effects of various lycopene production levels on acetic acid tolerance, lower lycopene production strains (yPS003, yPS004, and yPS005) were generated by adjusting the promoter strength of gene *crtE*. Nucleotide analog mutagenesis was used to generate a series of promoter mutants of varying strengths [31]. These promoter mutants were assembled with the *crtE* genes into a library of lycopene pathways with different expression patterns using the yeast assembly method [32]. In addition, higher lycopene production strains yPS006 and yPS007 were generated by incorporating another copy of lycopene pathway into yPS003 and yPS002, respectively. As shown in Fig. 2a, the lycopene yields of yPS007, yPS006, yPS002, yPS005, yPS003, yPS004, and yPS001 were 3.265, 2.311, 1.475, 1.322, 1.073, 0.759, and 0 mg/g DCW in the seed stage, respectively. Strains containing different lycopene produced during the aerobic fermentation of the seed stage were subjected to anaerobic fermentation at an initial optical density of 1.0 and acetic acid level of 5.5 g/L. As shown in Fig. 2b, the lag phases of yPS002, yPS005, yPS003, yPS004, yPS006, yPS007, and yPS001 were 12, 12, 24, 24, 24, 36, and 36 h, respectively. In addition, the highest cell density of yPS007 was 1.187-fold of yPS001. These results demonstrated that intracellular synthesis of lycopene in yeast could improve the tolerance to acetic acid. Moreover, it is indicated that yPS002, producing 1.475 mg/g DCW of lycopene, was the best strain in improving acetic acid tolerance in this study. Thus, it is important to regulate the endogenous concentration of lycopene to improve the tolerance of acetic acid to the yeast.

### Endogenous lycopene-enhanced ethanol fermentation

**Fig. 3:**
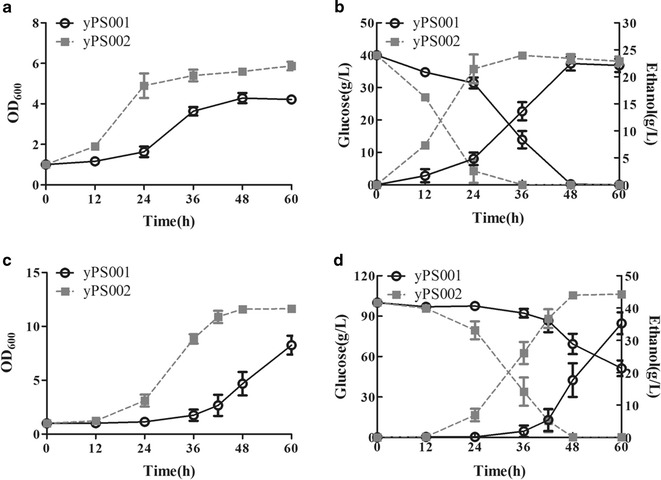
Effects of intracellular synthesis of lycopene on ethanol fermentation. **a**, **b** Fermentative profiles of two strains within 60 h (4% glucose, 1% yeast extract, 2% peptone and 5.5 g/L HAc) (pH = 4.05). **c**, **d** Fermentative profiles of two strains within 60 h (10% glucose, 1% yeast extract, 2% peptone and 5.5 g/L HAc) (pH = 4.05). Data are averages from three independent experiments (error bars represent SD)

To assay the performance of yeast containing the lycopene pathway during ethanol fermentation with acetic acid, yPS002, accumulating lycopene during the aerobic fermentation of the seed stage, was subjected to anaerobic fermentation at an initial optical density of 1.0 and acetic acid level of 5.5 g/L. The growth of cells was measured as optical density at specific intervals of time, within 60 h. As shown in Fig. 3 and Table 1, endogenous lycopene in yeast helped accelerate the process of ethanol fermentation under acetic acid and extremely reduce the lag phases in two groups. In the system of 40 g/L glucose, compared with yPS001, the highest cell density of yPS002 increased by 39%. Glucose consumption rate and ethanol production rate of yPS002 got to 1.49 and 0.67 g/L/h, respectively, which were 1.80- and 1.42-fold of the control strain. Evidently, there was a more strength in the group of fermentation with 100 g/L glucose. The lag phase was strongly reduced by intracellular synthesis of lycopene, which was 24 h shorter than that of control strain. The ethanol production was up to 44.25 g/L at 48 h; however, the control strain was just beginning to produce ethanol at that moment. The glucose consumption rate was 2.57-fold of that of yPS001. The above data demonstrated that intracellular synthesis of lycopene could increase acetic acid tolerance and ethanol production of yeast.

**Table 1:** Fermentative performance under acetic acid

	yPS001	yPS002	yPS001	yPS002
Initial glucose, g/L	40	40	100	100
Lag phase, h	12	0	12	36
Residual glucose, g/L	0	0	51.26 ± 5.86	0
Glucose consumption rate, g/L/h	0.83 ± 0.003	1.49 ± 0.15	0.81 ± 0.10	2.08 ± 0.003
Ethanol titer, g/L	22.45 ± 1.30	23.97 ± 0.25	35.29 ± 3.39	44.25 ± 0.38
Ethanol production rate, g/L/h	0.47 ± 0.03	0.67 ± 0.01	0.59 ± 0.06	0.92 ± 0.02

Consequently, lycopene overexpressed in *S. cerevisiae* shorted the lag period and accelerated the ethanol fermentation process. A key feature of strains with an enhanced tolerance is shorter lag period relative to control. We conclude that intracellular synthesis of lycopene could increase acetic acid tolerance. Better fermentative performance was observed in strain yPS002 at higher concentrations of initial glucose medium, which was consistent with the literature [33].

In addition, to test whether the production of lycopene increases the metabolic burdens or hampers yeast fitness, we assayed growth profiles of the control and lycopene producing strains without acetic acid stress. As shown in Additional file 1: Figure S2, there was no significant difference in cell growth in lycopene-expressing strains compared to the control strains without acetic acid, which indicated that strains expressing lycopene in this study did not increase the metabolic burdens. It is possible that the yield of lycopene in the strains yPS002 was low and caused no growth defect during anaerobic fermentation.

### Endogenous lycopene increasing oxidative stress resistance

**Fig. 4:**
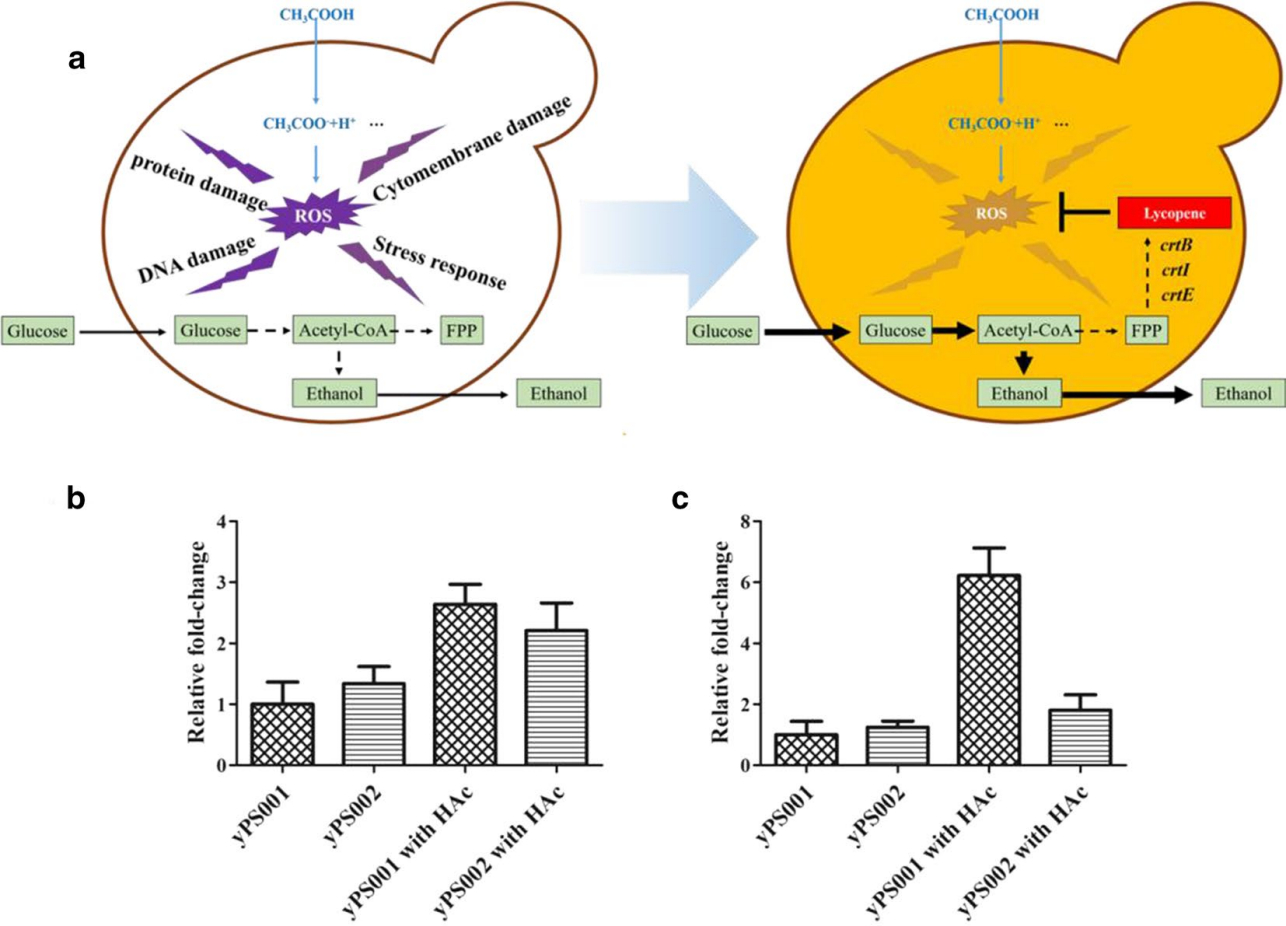
Endogenous lycopene increasing oxidative stress resistance. **a** Hypothesis on the mechanism of an enhanced tolerance to acetic acid in lycopene-expressing strains. **b** Results of changes in the transcription level of *CTT1* related to acetic acid stress in *S. cerevisiae* by RT-qPCR. **c** Results of changes in the transcription level of *INO1* related to acetic acid stress in *S. cerevisiae* by RT-qPCR. HAc was abbreviated from acetic acid in figures. Data are averages from three independent experiments (error bars represent SD)

In general, acetic acid affects cell metabolism and stabilities of proteins by a drop in intracellular pH and potential, leading to negative effects on cell growth and proliferation [34]. Acetic acid diffusing across plasma membrane damages cells by accumulating ROS [3], which can inhibit cell viability and ethanol production in *S. cerevisiae* [23]. ROS is formed upon incomplete reduction of oxygen and includes the hydroxyl radical (HO^−^), superoxide anion (O^2−^), and hydrogen peroxide (H_2_O_2_) [35], which cause protein damage, DNA damage, and membrane damage to the normal cells (Fig. 4a). Moreover, high oxidative stress up-regulated some stress response genes [36]. Therefore, reducing intracellular ROS level has become an available method for construction of acetic acid-tolerant strains. Lycopene, synthesized from FPP through *crtE*, *crtI,* and *crtB* in yeast [37], is a highly unsaturated hydrocarbon open chain [38]. Hence, it can reduce ROS level by trapping chain-carrying peroxyl radicals to enhance the tolerance of the strain to acetic acid. The ability of suppressing singlet oxygen from lycopene is two times more effective than β-carotene and ten times more potent than α-tocopherol [39–41]. Our experiments proved that lycopene can reduce the intracellular ROS level caused by acetic acid stress.

It was reported that lignocellulose-derived inhibitors can induce expression of oxidative stress-sensitive genes [36]. For example *CTT1*, encoding cytosolic catalase T, has a role in protection from oxidative damage by hydrogen peroxide [42]. Overexpression of *CTT1* can reduce cellular oxidative stress. Previous work in our lab demonstrated *INO1* is also an oxidative stress sensitive gene [28]. *INO1* encodes inositol-3-phosphate synthase involved in synthesis of inositol phosphates and inositol-containing phospholipids [43]. Up-regulation of *INO1* accelerated the membrane reconfiguration of cells to reduce oxidative damage. It is indicated that the expression of those genes is strongly associated with the intracellular oxidative stress. We, therefore, used the expression of the two genes as indicator for assaying the change of oxidative stress in lycopene production strains with acetic acid. As shown in Fig. 4b, c, the *CTT1* gene and *INO1* gene expressions of yPS001 with acetic acid were both significantly up-regulated than that of yPS001 without acetic acid, which were the same as other reports regarding yeast response to oxidative stress [19, 44]. However, the *CTT1* gene and *INO1* gene expressions of yPS002 with acetic acid decreased to 0.84- and 0.29-fold compared to that of yPS001 with acetic acid, respectively. It was indicated that  intracellular lycopene reduced intracellular ROS stress and thus decreased the transcription levels of *CTT1* and *INO1*. Our results confirmed that endogenous lycopene can decrease oxidative stress and, therefore, improve  intracellular environment.

As the previous literature indicated both phenol and furfural can induce ROS accumulated [45, 46], we further evaluated the effects of endogenous lycopene on other lignocellulose-derived inhibitors, like phenol and furfural (Additional file 1: Figures S3, S4). Compared to the control, growth advantages were observed in the lycopene-expressing strains in the stress of phenol or furfural. These results indicated that endogenous lycopene has a positive role in increasing the yeast tolerance under appropriate concentrations of phenol and furfural in different yeast chassis. Above all, our study gives a new strategy for reducing the ROS burden by accumulating intracellular antioxidant in *S. cerevisiae* during lignocellulosic fermentation.

## Conclusions

In this study, we illustrate that the intracellular ROS is associated with acetic acid stress in yeast. Incorporation of heterogeneous lycopene pathway in yeast can not only increase the cell viability via accumulating lycopene for antioxidant, but also improve the ethanol production level during anaerobic fermentation. Meanwhile, we developed a serial yeast strains producing different levels of lycopene and proved the strains with 1.475 mg/g DCW lycopene production have significant growth advantages. We demonstrate that endogenous lycopene can decrease intracellular ROS level caused by acetic acid, thus increasing cell growth and ethanol production. This work innovatively investigates a new strategy for second generation bioethanol production during lignocellulosic fermentation.

## Methods

### Strains, media, and plasmids

All the *S. cerevisiae* strains used in this study are listed in Table 2. *S. cerevisiae* strains were cultivated at 30 °C in YPD medium (1% (w/v) yeast extract, 2% (w/v) peptone, and 2% (w/v) glucose). The engineered yeast cells were selected and grown on synthetic dextrose (SD) medium containing 6.7 g/L yeast nitrogen base without amino acids, 2 g/L complete supplement mixture (without histidine, leucine, tryptophan, uracil), 20 g/L glucose, 20 mg/L histidine, 20 mg/L tryptophan, and 20 mg/L uracil (SD-Leu), or SD medium containing 6.7 g/L yeast nitrogen base without amino acids, 2 g/L complete supplement mixture (without histidine, leucine, tryptophan, uracil), 20 g/L glucose, 20 mg/L tryptophan, and 20 mg/L uracil (SD-Leu-His).

**Table 2:** Plasmids and strains used in this study

Name	Description	Source
Plasmids		
pCaro415	pTEF1-crtE-tPDX1-pTDH3-crtI-tMPE1pFBA1-crtB-tTDH2, pRS415	This study
pCaro-2	pTEF1(mutagenized-M1)-crtE-tPDX1-pTDH3-crtI-tMPE1pFBA1-crtB-tTDH2, pRS415	This study
pCaro-3	pTEF1(mutagenized-M2)-crtE-tPDX1-pTDH3-crtI-tMPE1pFBA1-crtB-tTDH2, pRS415	This study
pCaro-4	pTEF1(mutagenized-M3)-crtE-tPDX1-pTDH3-crtI-tMPE1pFBA1-crtB-tTDH2, pRS415	This study
pCaro413	pTEF1-crtE-tPDX1-pTDH3-crtI-tMPE1pFBA1-crtB- tTDH2, pRS413	This study
Strains		
CEN.PK2-1C	*MATa*, ura3-52, trp1-289, leu2-3, 112, his3Δ1, MAL2-8C, SUC2	EUROSCARF
BY4741	*MATa*, his3Δ1, leu2Δ0, LYS2, met15Δ0, ura3Δ0	EUROSCARF
BY4742	*MATα*, his3Δ1, leu2Δ0, lys2Δ0, MET15, ura3Δ0	EUROSCARF
yPS001	CEN.PK2-1C, pRS415	This study
yPS002	CEN.PK2-1C, pCaro415	This study
yPS003	CEN.PK2-1C, pCaro-2	This study
yPS004	CEN.PK2-1C, pCaro-3	This study
yPS005	CEN.PK2-1C, pCaro-4	This study
yPS006	yPS003, pCaro413	This study
yPS007	yPS002, pCaro413	This study
yPS008	BY4741, pRS415	This study
yPS009	BY4741, pCaro415	This study
yPS010	BY4742, pRS415	This study
yPS011	BY4742, pCaro415	This study

Plasmid cloning work and circuit construct characterization were both performed in *Escherichia coli* DH10B strains, which were cultured in LB (Luria–Bertani Broth) media (1% (w/v) peptone, 0.5% (w/v) NaCl, and 0.5% (w/v) yeast extract). The pCaro415 and pCaro413 were constructed using yeast assemble method. Specifically, The TEF1p, tTDH3p, tFBA1p, and TDH2t were PCR amplified from genome of *S. cerevisiae*. The *crtE*, *crtI,* and *crtB* were PCR amplified from the Registry of Standard Biological Parts (http://partsregistry.org). The library of mutagenized TEF1 promoter was generated via error-prone PCR with primers TEF1-F (5′-CTCACTATAGGGCGAATTGGGTACCGGGCCCCCCCTCGAG-3′) and TEF1-R (5′-ACTCGAGTGGAATTGCTGTGAGGATGTTCGCGTAATCCAT-3′) using pCaro415 as the template. About 0.8 mM Mn^2+^ was added to the Taq DNA polymerase buffer (Tiangen, China) to achieve the highest mutation rate and reduce the ratio of multiple mutants in one DNA fragment. The mutagenized TEF1 promoter and the PCR fragment of crtE-PDX1t-TDH3p-crtI-MPR1t- FBA1p-crtB-TDH2t and the pRS415 vector digested with *Bam*HI and *Sal*I were co-transformed to CEN.PK2-1C for yeast assemble. Colonies with lighter color were selected for fermentation analysis.

### Reactive oxygen species analysis

The ROS levels were measured according to the reported method [47] with some modifications in which the two acetate groups of 2′7′-dichlorofluorescein diacetate (DCFH-DA) were cleaved by the intracellular esterase to yield 2′,7′-dichlorofluorescein (DCFH), and the DCFH was then oxidized by ROS resulting in forming the highly fluorescent 2′,7′-dichlorofluorescein (DCF). Briefly, the fermentation broth at 12 h containing 10^7^ cells was centrifuged at 4 °C, washed twice with phosphate buffer (PBS, pH = 7.0), and resuspended in 1 mL PBS. Then, adding 10 μg of DCFH-DA (2.5 mg/mL of stock solution dissolved in DMSO) and incubated at 30 °C for 60 min. After the reaction, the broth was centrifuged for 5 min at 4 °C and 5000*g*. The cell pellets were washed twice with 1 mL PBS, and resuspended in 1 mL PBS. The fluorescence intensity was observed by Olympus CX41 fluorescence microscope, and the fluorescence was measured in a multimode plate reader (SpectraMax M2, Molecular Devices, USA) at excitation wavelength of 488 nm and emission wavelength of 525 nm. The whole process of analysis was carried out in dark. Diverse intensities of the fluorescence were transformed into electronic signals by the apparatus, and the data were given in relative fluorescence intensity. Colony-forming units (CFU) were measured with flat colony counting method. The fluorescence intensity per cell was used to represent the intracellular ROS levels. The DNA sequence of *TEF1* promoter and the different mutants can be found in Additional File 2.

### Acetic acid, phenol, and furfural tolerance assay

To examine the phenotype of endogenous expression of lycopene, yPS001 and yPS002 were cultured in SD-Leu media until the late log phase, and serially diluted cells were spotted onto YPD agar plates containing acetic acid. Approximately 10^6^ cells and serial dilutions of 10^−1^–10^−5^ (from left to right) of strains were spotted on YPD plates with inhibitors. Plates were incubated at 30 °C for 48 h.

### Anaerobic fermentation

Colonies on solid plates were picked up and cultured in 5 mL SD-Leu or SD-Leu-His medium and grown at 30 °C, 250 rpm for 24 h to exponential phase (OD_600_ = 8.0). Then, the preculture was transferred into 50 mL fresh SD-Leu or SD-Leu-His medium for further 24 h cultivation (to OD_600_ = 10.0). Then, the seed culture was transferred into a 250 mL shake-flask containing 50 mL fermentation medium (4% glucose, 1% yeast extract, and 2% peptone), or 100 mL fermentation medium (10% glucose, 1% yeast extract, and 2% peptone) with an initial OD_600_ of 1.0 and cultivated at 30 °C, 150 rpm for 60 h.

### Analysis of cell growth, sugars, and ethanol

At designated time, the fermentation broth was taken using syringe for further analysis. To analyze the cell concentration, the optical densities were measured at 600 nm (TU-1810 UV spectrophotometer, Persee, China).

1 mL fermentation broth was taken by syringe and centrifuged immediately at 12,000 rpm for 5 min. Before analysis, the supernatant was filtered with a 0.22 μm filters to remove impurities and stored at − 80 °C. The concentration of glucose and ethanol was analyzed using HPLC (Waters Corp., USA) with a Aminex HP-87H column (Bio-Rad, Hercules, CA, USA) at 65 °C. The mobile phase and its flow rate were 5 mM H_2_SO_4_ and 0.6 mL/min, respectively [48].

### Extraction and analysis of lycopene

Extraction of lycopene was as described by Chen et al. [49] with some modifications. Briefly, cells harvested from cultures were washed, resuspended in boiling 3 N HCl for 3 min, and cooled in an ice-bath for 3 min. Then, cells debris were washed twice with water, resuspended in acetone containing 0.1% BHT (w/v), vortexed until colorless, and followed by centrifugation. The acetone phase containing the extracted lycopene was filtered for HPLC analysis. An HPLC system (Waters e2695) equipped with a BDS Hypersil C18 column (5.0 × 2.1 mm, 2.7 µm) and a UV/VIS detector (Waters 2489) was used to analyze the produced lycopene. The signal of lycopene was detected 470 nm. The mobile phase consisted of methanol–acetonitrile–dichloromethane (9:40:1 v/v) with a flow rate of 0.3 mL/min at 22 °C.

### Real-time reverse transcription-PCR

Real-time reverse transcription-PCR (RT-PCR) was as described by Wu et al. [50] with some modifications. The total RNA was extracted from yeast cells by Trizol. Then, complementary DNA (cDNA) was generated from isolated RNA using the TransScript First-Strand cDNA Synthesis Kit (Trans, China). Converted cDNA was added to Top/Tip Green qRCR SuperMix and specific Primer and subjected to RT-PCR analysis employing the CFX96 Cycler-Real-Time PCR Detection System (Bio-Rad Laboratories, Inc., Hercules, CA, USA), in white-walled PCR plates (96 wells). The cycle conditions were set as follows: initial template denaturation at 94 °C for 3 min, followed by 45 cycles of denaturation at 94 °C for 5 s, and combined primer annealing/elongation at 60 °C for 30 s. The primer sequences used in the experiment are shown in Additional file 3: Table S1, and ALG9 was selected as the internal Ref. [51] .

## Additional files

**Additional file 1: Figure S1.** Stress response of lycopene expression in BY4741 or BY4742 to the presence of acetic acid by serial dilution assay. **Figure S2.** Fermentative profiles of yPS001 and yPS002 within 60 h during anaerobic fermentation without acetic acid. **Figure S3.** Stress response of lycopene expression in CEN.PK2-1C to the presence of phenol or furfural by serial dilution assay. **Figure S4.** Stress response of lycopene expression in BY4741 or BY4742 to the presence of phenol or furfural by serial dilution assay.

**Additional file 2: ** Supporting Online Text: DNA sequences of *TEF1* promoter and the different mutants obtained in this work.

**Additional file 3.** Table S1. The primers for RT-qPCR analysis.

## Abbreviations

*S. cerevisiae*: *Saccharomyces cerevisiae*
ROS: reactive oxygen species
HAc: acetic acid
LB: Luria–Bertani Broth
SD: synthetic complete drop-out medium
SD-Leu: SD medium without histidine
SD-Leu-His: SD medium without leucine and histidine
DCFH-DA: 2′,7′-dichlorofluorescein diacetate
DCFH: 2′,7′-dichlorofluorescein
DCF: 2′,7′-dichlorofluorescein
PBS: phosphate buffer
CFU: colony-forming units
cDNA: complementary DNA
RT-PCR: real-time reverse transcription-PCR
