# Supplementary material for: Endogenous lycopene improves ethanol production under acetic acid stress in Saccharomyces cerevisiae
Source: Biotechnol Biofuels. 2018 Apr 10;11:107. doi: 10.1186/s13068-018-1107-y (PMC5891932; doi:10.1186/s13068-018-1107-y)
Supplement: Supplementary file 1 — Additional file 1: Figure S1. Stress response of lycopene expression in BY4741 or BY4742 to the presence of acetic acid by serial dilution assay. Figure S2. Fermentative profiles of yPS001 and yPS002 within 60 h during anaerobic fermentation without acetic acid. Figure S3. Stress response of lycopene expression in CEN.PK2-1C to the presence of phenol or furfural by serial dilution assay. Figure S4. Stress response of lycopene expression in BY4741 or BY4742 to the presence of phenol or furfural by serial dilution assay. [file 13068_2018_1107_MOESM1_ESM.docx]

**Additional Information:**

**Endogenous lycopene improves ethanol production under acetic acid stress in *Saccharomyces cerevisiae***

Shuo Pan^1,2^†, Bin Jia^1,2^†, Hong Liu^1,2^, Zhen Wang^1,2^, Meng-Zhe Chai^1,2^, Ming-Zhu Ding^1,2^, Xiao Zhou^1,2^, Xia Li^1,2^, Chun Li^1^, Bing-Zhi Li^1,2^, Ying-Jin Yuan^1,2^*

^1^Key Laboratory of Systems Bioengineering (Ministry of Education), School of Chemical Engineering and Technology, Tianjin University, Tianjin, 300072, PR China

^2^SynBio Research Platform, Collaborative Innovation Center of Chemical Science and Engineering (Tianjin), Tianjin University, Tianjin, 300072, PR China

*Corresponding author: Y-J Yuan, E-mail: yjyuan@tju.edu.cn; Tel: 86-22-27403888

Fax: 86-22-27403389

†Equal contribution.

**Additional file 1**


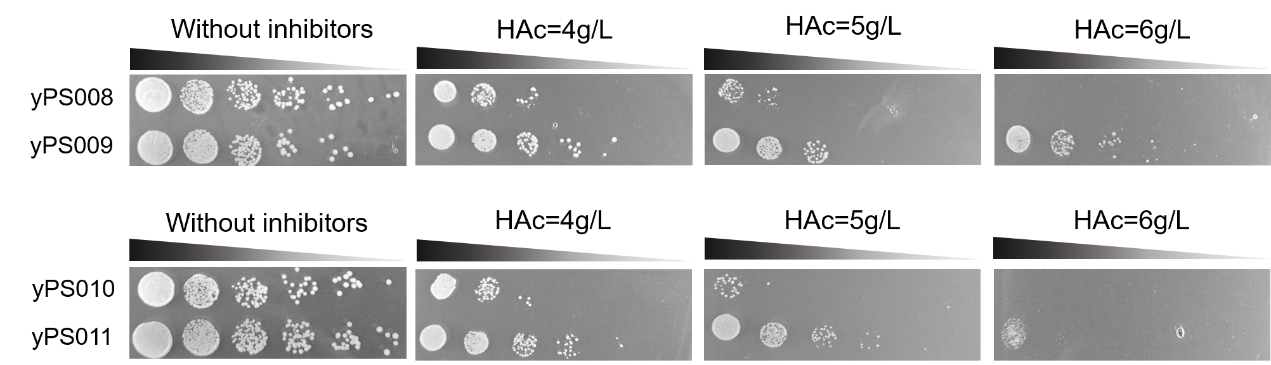


**Figure S1. Stress response of lycopene expression in BY4741 or BY4742 to the presence of acetic acid by serial dilution assay.** BY4741 with pRS415 (yPS008), BY4741 with pathway of lycopene (yPS009), BY4742 with pRS415 (yPS010) and BY4742 with pathway of lycopene (yPS011) were cultured in SD-Leu media until the late log phase, and serially diluted cells were spotted onto YPD agar plates containing acetic acid. Approximately 10^6^ cells and serial dilutions of 10^-1^ to 10^-5^ (from left to right) of strains were spotted on YPD plates with inhibitors. Plates were incubated at 30°C for 48h. HAc was abbreviated from acetic acid in figures.

.


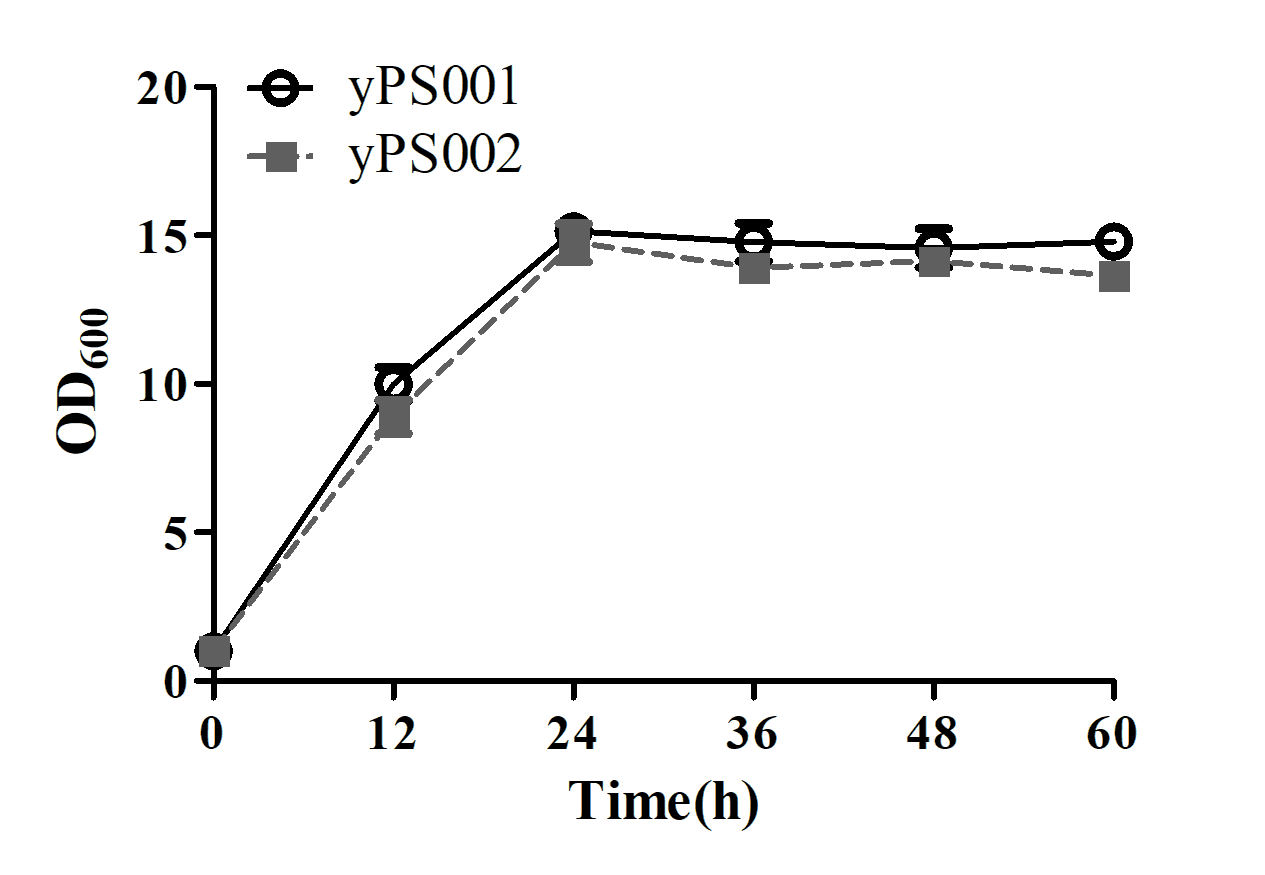


**Figure S2. Fermentative profiles of yPS001 and yPS002 within 60 h in fermentation medium without acetic acid (10% glucose, 1% yeast extract, 2% peptone)**. CEN.PK2-1C with pRS415 (yPS001) and CEN.PK2-1C with pathway of lycopene (yPS002) were subjected to anaerobic fermentation at an initial optical density of 1.0. Data are averages from three independent experiments (error bars represent SD).


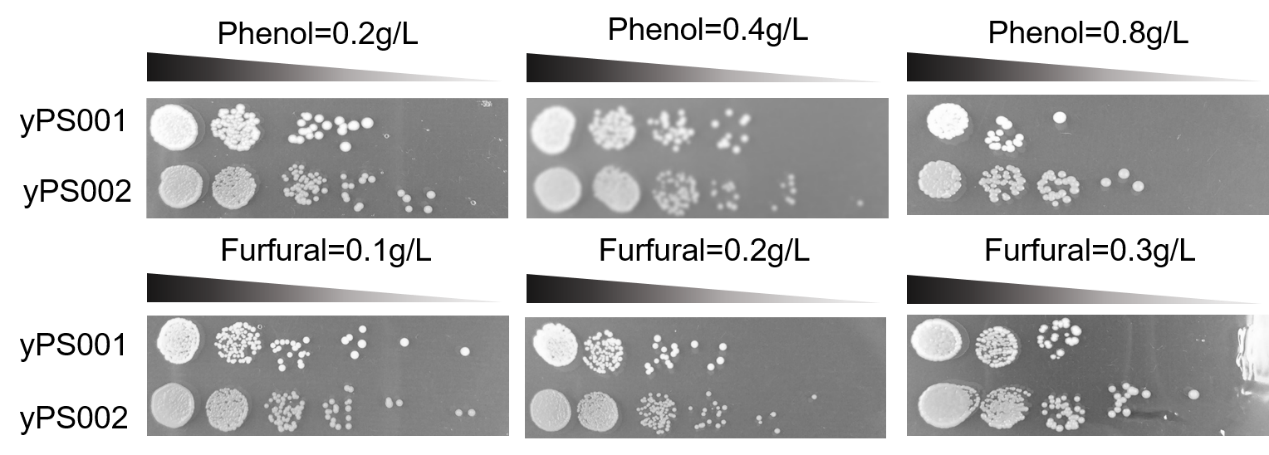


**Figure S3. Stress response of lycopene expression in CENPK2-1C to the presence of phenol or furfural by serial dilution assay.** CEN.PK2-1C with pRS415 (yPS001) and CEN.PK2-1Cwith pathway of lycopenewere (yPS002) cultured in SD-Leu media until the late log phase, and serially diluted cells were spotted onto YPD agar plates containing phenol or furfural. Approximately 10^6^ cells and serial dilutions of 10^-1^ to 10^-5^ (from left to right) of strains were spotted on YPD plates with inhibitors. Plates were incubated at 30°C for 48h.


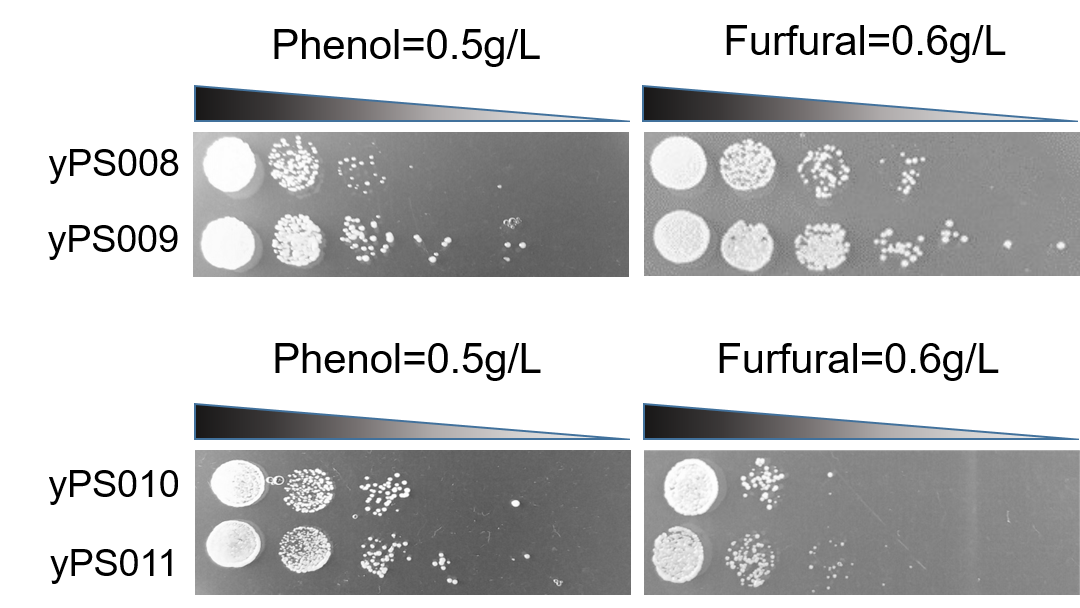


**Figure S4. Stress response of lycopene expression in BY4741 or BY4742 to the presence of phenol or furfural by serial dilution assay.** BY4741 with pRS415 (yPS008), BY4741 with pathway of lycopene (yPS009), BY4742 with pRS415 (yPS010) and BY4742 with pathway of lycopene (yPS011) were cultured in SD-Leu media until the late log phase, and serially diluted cells were spotted onto YPD agar plates containing phenol or furfural. Approximately 10^6^ cells and serial dilutions of 10^-1^ to 10^-5^ (from left to right) of strains were spotted on YPD plates with inhibitors. Plates were incubated at 30°C for 48h.
