# Supplementary material for: Endogenous lycopene improves ethanol production under acetic acid stress in Saccharomyces cerevisiae
Source: Biotechnol Biofuels. 2018 Apr 10;11:107. doi: 10.1186/s13068-018-1107-y (PMC5891932; doi:10.1186/s13068-018-1107-y)
Supplement: Supplementary file 2 — Additional file 2: Supporting Online Text: DNA sequences of TEF1 promoter and the different mutants obtained in this work. [file 13068_2018_1107_MOESM2_ESM.docx]

**Additional Information:**

**Endogenous lycopene improves ethanol production under acetic acid stress in *Saccharomyces cerevisiae***

Shuo Pan^1,2^†, Bin Jia^1,2^†, Hong Liu^1,2^, Zhen Wang^1,2^, Meng-Zhe Chai^1,2^, Ming-Zhu Ding^1,2^, Xiao Zhou^1,2^, Xia Li^1,2^, Chun Li^1^, Bing-Zhi Li^1,2^, Ying-Jin Yuan^1,2^*

^1^Key Laboratory of Systems Bioengineering (Ministry of Education), School of Chemical Engineering and Technology, Tianjin University, Tianjin, 300072, PR China

^2^SynBio Research Platform, Collaborative Innovation Center of Chemical Science and Engineering (Tianjin), Tianjin University, Tianjin, 300072, PR China

*Corresponding author: Y-J Yuan, E-mail: yjyuan@tju.edu.cn; Tel: 86-22-27403888

Fax: 86-22-27403389

†Equal contribution.

**Additional file 2**

**Supporting Online Text: DNA Sequences of *TEF1* promoter and the different mutants obtained in this work.**

a) DNA sequence of the wild-type *pTEF1*

CCACACACCATAGCTTCAAAATGTTTCTACTCCTTTTTTACTCTTCCAGATTTTCTCGGACTCCGCGCATCGCCGTACCACTTCAAAACACCCAAGCACAGCATACTAAATTTCCCCTCTTTCTTCCTCTAGGGTGTCGTTAATTACCCGTACTAAAGGTTTGGAAAAGAAAAAAGAGACCGCCTCGTTTCTTTTTCTTCGTCGAAAAAGGCAATAAAAATTTTTATCACGTTTCTTTTTCTTGAAAATTTTTTTTTTGATTTTTTTCTCTTTCGATGACCTCCCATTGATATTTAAGTTAATAAACGGTCTTCAATTTCTCAAGTTTCAGTTTCATTTTTCTTGTTCTATTACAACTTTTTTTACTTCTTGCTCATTAGAAAGAAAGCATAGCAATCTAATCTAAGTTTTAATTACAAA

b) DNA sequence of *pTEF1* (mutagenized-M1)

CCACACACCATAGCTTCAAAATGTTTCTACTCCTTTTTTACTCTTCCAGATTTTCTCGGACTCCGCGCATCGCCGTACCACTTCAAAACACCCAAGCACAGCATACTAAATTTCCCCTCTTTCTTCCTCTAGGGTGTCGTTAATTACCCGTACTAAAGGTTTGGAAAAGAAAAAAGAGACCGCCTCGTTTCTTTTTCTTCGTCGAAAAAGGCAATAAAAATTTTTATCACGTTTCTTTTTCTTGAAAATTTTTTTTTTGATTTTTTTCTCTTTCGATGACCTCCCATTTGATATTTAAGTTAATAAACGGTCTTCAATTTCTCAAGTTTCAGTTTCATTTTTCTTGTTCTATTACAACTTTTTTTACTTCTTGCTCATTAGAAAGAAAGCATAGCAATCTAATCTAAGTTTTAATTACAAA

c) DNA sequence of *pTEF1* (mutagenized-M2)

CCACACACCATAGCTTCAAAATGTTTCTACTCCTTTTTTACTCTTCCAGATTTTCTCGGACTCCGCGCATCGCCGTACCACTTCAAAACACCCAAGCACAGCATACTAAATTTCCCCTCTTTCTTCCTCTAGGGTGTCGTTAATTACCCGTACTAAAGGTTTGGAAAAGAAAAAAGAGACCGCCTCGTTTCTTTTTCTTCGTCGAAAAAGGCAATAAAAATTTTTATCACGTTTCTTTTTCTTGAAAATTTTTTTTTTGATTTTTTTCTCTTTCGATGACCTCCCATTGATATTTAAGTTAATAAACGGTCTTCAATTTCTCAAGTTTCAGTTTCATTTTTCTTGTTCTATTACAACTTTTTTTACTTCTTGCTCATTAGAAAGAAAGCATGGCAATCTAATCTAAGTTTTAATTACAAA

d) DNA sequence of *pTEF1* (mutagenized-M3)

CCACACACCATAGCTTCAAAATGTTTCTACTCCTTTTTTACTCTTCCAGATTTTCTCGGACTCCGCGCATCGCCGTACCACTTCAAAACACCCAAGCACAGCATACTAAATTTCCCCTCTTTCTTCCTCTAGGGTGTCGTTAATTACCCGTACTAAAGGTTTGGAAAAGAAAAAAGAGACCGCCTCGTTTCTTTTTCTTCGTCGAAAAAGGCAATAAAAATTTTTATCACGTTTCTTTTTCTTGAAAATTTTTTTTTTGATTTTTTTCTCTTTCGATGACCTCCCATTGATATTTAAGTTAATAAACGGTCTTCAATTTCTCAAGTTTCAGTTTCATTTTTCTTGTTCTATTACAACTTTTTTTACTTCTTGCTCATTAGAAAGAAAGCATAGCAATCTAATCTAAGTTTCTCATACGAA
