# Supplementary material for: Endogenous lycopene improves ethanol production under acetic acid stress in Saccharomyces cerevisiae
Source: Biotechnol Biofuels. 2018 Apr 10;11:107. doi: 10.1186/s13068-018-1107-y (PMC5891932; doi:10.1186/s13068-018-1107-y)
Supplement: Supplementary file 3 — Additional file 3. Table S1. The primers for RT-qPCR analysis. [file 13068_2018_1107_MOESM3_ESM.docx]

**Additional Information:**

**Endogenous lycopene improves ethanol production under acetic acid stress in *Saccharomyces cerevisiae***

Shuo Pan^1,2^†, Bin Jia^1,2^†, Hong Liu^1,2^, Zhen Wang^1,2^, Meng-Zhe Chai^1,2^, Ming-Zhu Ding^1,2^, Xiao Zhou^1,2^, Xia Li^1,2^, Chun Li^1^, Bing-Zhi Li^1,2^, Ying-Jin Yuan^1,2^*

^1^Key Laboratory of Systems Bioengineering (Ministry of Education), School of Chemical Engineering and Technology, Tianjin University, Tianjin, 300072, PR China

^2^SynBio Research Platform, Collaborative Innovation Center of Chemical Science and Engineering (Tianjin), Tianjin University, Tianjin, 300072, PR China

*Corresponding author: Y-J Yuan, E-mail: yjyuan@tju.edu.cn; Tel: 86-22-27403888

Fax: 86-22-27403389

†Equal contribution.

**Additional file 3**

**Table S1 The primers for RT-qPCR analysis**

| **Gene** | **Primer name** | **Primer (5’→3’)** |
| --- | --- | --- |
| ALG9 | rtALG9-F | CATGGCAACGGCAGAAGGCAATAA |
|  | rtALG9-R | ATCGTGAAATTGCAGGCAGCTTGG |
| CTT1 | rtCTT1-F | CGTTGGTGGTGAAAGTGGTA |
|  | rtCTT1-R | TCTGAGGAAGAAGACGGGAGT |
| INO1 | rtINO1-F | GGCTTCACCAAGGACATCTT |
|  | rtINO1-R | CAAACGACTTTGTCGTCTCTG |
